# Psychometric properties of the Diabetes Self-Management Questionnaire (DSMQ) in Urdu

**DOI:** 10.1186/s12955-017-0776-8

**Published:** 2017-10-12

**Authors:** Allah Bukhsh, Shaun Wen Huey Lee, Priyia Pusparajah, Andreas Schmitt, Tahir Mehmood Khan

**Affiliations:** 1 School of Pharmacy, Monash University, Jalan Lagoon Selatan, 47500 Bandar Sunway, Selangor Malaysia; 2 Institute of Pharmaceutical Sciences, University of Veterinary & Animal Sciences, 54000 Lahore, Pakistan; 3 Asian Centre for Evidence Synthesis in Population, Implementation and Clinical Outcomes (PICO), Health and Well-being Cluster, Global Asia in the 21st Century (GA21) Platform, Monash University Malaysia, Bandar Sunway, Selangor Malaysia; 4 Jeffrey Cheah School of Medicine and Health Sciences, Monash University, Jalan Lagoon Selatan, 47500 Bandar Sunway, Selangor Malaysia; 5 Research Institute of the Diabetes Academy Mergentheim (FIDAM), German Diabetes Center Mergentheim, Theodor-Klotzbücher-Str. 12, 97980 Bad Mergentheim, Germany

**Keywords:** Self-care, Type 2 diabetes, Validation, HbA1c, Psychometric analysis

## Abstract

**Background:**

Numerous study tools on diabetes self-care have been introduced; however, most existing tools do not show expectable and meaningful correlations with patients’ glycaemic control. The Diabetes Self-Management Questionnaire (DSMQ) was designed to appraise self-care activities which can predict glycaemic control outcomes. However, this tool has not been validated in Pakistan. Therefore, the aim of this study was to translate and examine the psychometric properties of the Urdu version of DSMQ among type 2 diabetes patients.

**Method:**

Standard forward-backward translation was used to translate the DSMQ into Urdu language. A convenience sample of 130 patients with type 2 diabetes was collected to assess the Urdu version’s psychometric properties. Reliability was assessed by Cronbach’s coefficient α and validity was assessed using confirmatory factor analysis and criterion-related correlations.

**Results:**

High internal consistency was found for all DSMQ scales (Sum scale: α = 0.96, Glucose Management: 0.91; Dietary Control: 0.88; Physical Activity: 0.89; Health-Care Use: 0.73). The DSMQ subscales showed significant correlations with HbA1c (Glucose Management: −0.75; Dietary Control: −0.76; Physical Activity: −0.71; Health-Care Use: −0.64; Sum Scale: −0.78; all *p* < 0.001). However, when associations with HbA1c were assessed in one multiple linear regression model, only Glucose Management and Dietary Control were significantly associated with lower HbA1c values (Beta = −0.42, *p* = 0.004 and Beta = −0.30, *p* = 0.028, respectively), while Physical Activity and Health-Care Use were not (*p* > 0.05). Adequate fit to the data was achieved for single factor model after successively modelling all significant correlations between the items’ error terms, with Chi^2^ = 106.6, df = 84, *p* = 0.049; TLI = 0.98, CFI = 0.99 and RMSEA = 0.05 (90% CI 0.01–0.07). Whereas a comparatively lower fit indices to data were observed in case of four factor model.

**Conclusion:**

The findings support the Urdu version of the DSMQ as a reliable and valid instrument for assessing self-care activities associated with glycaemic control in type 2 diabetes patients.

**Electronic supplementary material:**

The online version of this article (10.1186/s12955-017-0776-8) contains supplementary material, which is available to authorized users.

## Background

By the most recent estimates, worldwide around 415 million people have been diagnosed with diabetes mellitus (DM), and the number is expected to increase to 642 million by 2040 [1], making it one of the leading non communicable health care problem worldwide [2]. Pakistan has been ranked 7th in diabetes disease burden in the world with prevalence rate of 7.6% to 11% in 2011, and it is projected to reach 15% (14 million) by year 2030. If the present development continues, Pakistan is expected to move to top 4th place [3]. The morbidity and mortality resulting from micro- and macro-vascular complications of type 2 DM continue to contribute a significant economic burden on individual patient as well as on society [4]. Glycosylated haemoglobin (HbA1c) is a major predictor of the future development of diabetes-related late complications, and by optimising glycaemic control the risks of such complications can be significantly reduced [5, 6].

Although there are many factors which can influence optimum glycemic control, adherence to relevant self-care behaviours such as healthy diet, regular exercise, self-monitoring of blood glucose and use of medication are considered to play the leading role in establishing euglycaemia [7–9]. Thus, to identify reasons for suboptimal glycaemic control, the assessment of patients’ self-care behaviours may be required. Therefore, a standardised self-care assessment tool could be of a great value for researchers and clinicians seeking to evaluate multiple domains of diabetes patient’s self-care [10–12].

To date various instruments have been used to examine diabetes patients’ self-care behaviours [13–21]. A recent systematic review on tools used to assess self-care behaviours in type 2 DM identified thirty different tools for this purpose. Out of those, 21 addressed multiple domains of diabetes self-care behaviours, and majority of tools were developed during last decade [22]. However, out of the 21 scales addressing multiple domains, only 10 were available in English language, and none was available in Urdu language. Furthermore, only few instruments showed psychometrically satisfactory properties. Additionally, weak associations with glycemic outcomes are the most frequently observed grey area for many of these tools. Another systematic review of psychometric tools spotted five study tools evaluating self-care, but only the Summary of Diabetes Self-Care Activities Measure (SDSCA) fully met the reviewers’ evaluation criteria. Although the SDSCA is the most commonly used instrument for diabetes related self-care activities, several studies did not support significant associations of this tool with Hb1Ac [10].

The Diabetes Self-Management Questionnaire (DSMQ) is a relatively new psychometric instrument to examine diabetes self-care activities [23]. The scale may be effective for clinical evaluation of patients with poor diabetes outcomes. Additionally, it could be of value for studies analysing the factors leading to poor self-care behaviours and glycemic control in diabetes patients. The primary objective when the DSMQ was developed, was to evaluate self-care behaviours in relation to HbA1c, so that the results would be applicable for mediational analyses. Additionally, the brief but comprehensive structure of the tool make it well suitable for clinical studies [24]. There is no validated tool in Pakistani native Urdu language to access self-care behaviours in diabetes patients. Although DSMQ is based on descriptors which satisfies its linguistic adaptation and validation into language and culture in which it was initially developed [23], but a cross-border and cross-cultural study is required for adaption of the questionnaire to suit regional characteristics and show its content validity and measurement properties equivalent to the original version [25, 26]. In countries with different languages and cultures, not only translation into new languages is required, but there is also a need for adaption of questionnaire to suit regional characteristics. Therefore, we translated the DSMQ into Urdu language using standard translation method. This article illustrates the DSMQ’s translation into Urdu language and the evaluation of its psychometric properties in Pakistani patients with type 2 DM.

## Methods

### Study design

A Cross-sectional study design was adopted for the data collection. A target sample size of 160 patients with type 2 DM was estimated based on the number of items to participant ratio of 1:10 [27]. The data collection was carried out from July to September 2016 at a tertiary hospital (Akhuwat Hospital) in Lahore, Pakistan, and Awan Medical Complex.

### Instrument translation

**Fig. 1:**
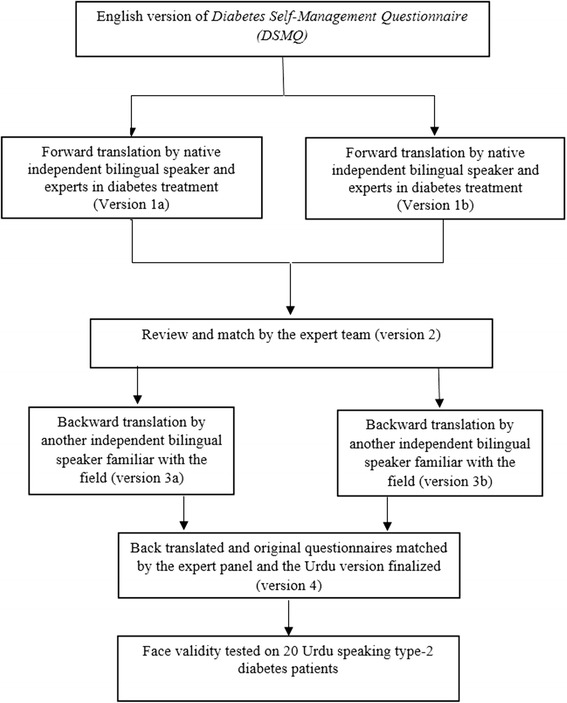
Translation of the Diabetes Self-Management Questionnaire (DSMQ): from English to Urdu

The English version of the DSMQ was translated into Urdu using a standardised forward and backward translation procedure, as recommended by Bradley [28] (Fig. 1). The DSMQ was developed to evaluate diabetes self-care behaviours associated with glycaemic control within common therapeutic options for adult patients of type 1 and type 2 DM. Respondents were asked to rate the 16 statements of DSMQ, describing specific self-care behaviours according to their own diabetes self-care during the last 2 months. Rating is done using on a four point Likert scale (from 0 – ‘does not apply to me’ to 3 – ‘applies to me very much’; a neutral response option is not available in order to extract more specific results). The tool comprises of seven positively keyed statements and nine negatively keyed statements (formulated inversely with respect to effective self-care behaviours).

In addition to a ‘Sum Scale’ scoring (summation of all 16 items score), the tool also enables the evaluation of four subscales’ score of diabetes self-care; namely ‘Glucose Management’(GM), which is comprised of five statements: 1, 4, 6, 10, 12, ‘Dietary Control’(DC): comprised of four statements: 2, 5, 9, 13, ‘Physical Activity’ (PA): comprised of three statements: 8, 11, 15, and, ‘Health-Care Use’ (HU): comprised of three statements: 3, 7, 14. The last item (item 16) asks the respondents to rate their overall diabetes self-care, hence its score is included only in the ‘Sum Scale’.

### Scoring criteria

The scoring of the DSMQ involves summing up the scores of all items after reversing the scores of nine negatively keyed statements (so that higher scores represent more effective self-care). The scale scores are then transformed to a scale ranging from 0 to 10, where a score of 10 indicates the most effective self-care behaviour.

### Participants and setting

Inclusion criteria for the study were adult age, type 2 DM, diagnosed at least 1 year before, recent HbA1c lab test (not more than 8 weeks older from the date of interview), taking hypoglycemic medications and sufficient communication skills in the Urdu language. Patients with terminal illness, cognitive impairments or inability to complete the interviews were excluded. Study participants were interviewed face-to-face for collection of socio-demographic data. Diabetes self-care activities were assessed using the translated (Urdu) version of the DSMQ. One hundred and thirty eligible patients consented to participate in the study (response rate approximately 81.3%). Patients’ medical records were reviewed by the investigator on the same day for HbA1c levels, type and number of hypoglycemic agents and presence of co-morbid conditions.

### Ethics approval

Ethical approval was obtained from the Monash University Human Research Ethics Committee (Approval no. 7767). Written consent was obtained from all study participants after providing them an oral and written information about the purpose and required procedure of the study.

### Statistical analysis

The analyses were performed using SPSS 21.0.0 (SPSS Inc., Chicago, IL, USA). Frequencies and descriptive statistics were used for patients’ demographic presentation, while means and standard deviations were calculated for the continuous variables.

### Reliability analysis

Reliability of the DSMQ was measured by using internal consistency. Cronbach’s α coefficient used to assess internal consistency, is appraised by the following criteria: > 0.9 = Excellent, > 0.8 = Good, > 0.70 = Acceptable, > 0.6 = Questionable, > 0.5 = Poor and <0.5 = Unacceptable [29]. Item characteristics were evaluated by corrected item-total correlations, corrected item-subscale correlations, potential increases of the scale’s reliability coefficient (Cronbach’s α) in case of item deletion, and, the item’s correlation with the HbA1c value [30, 31]. The scale was considered reliable when all items correlate with the total. An item showing poor correlation (<0.3) was considered for exclusion [32]. Pearson correlation (two-tailed test) was applied to HbA1c and DSMQ sum scores.

### Known group validity analysis

One-way Analysis of Variance was applied for known group’s validation after categorising the participants into three groups according to their HbA1c values. Patients’ glycaemic control was classified on the basis of HbA1c values into ‘good glycaemic control’ (HbA1c values ≤7.5%), ‘medium glycaemic control’ (HbA1c values ranging from 7.6% to 8.9%) and ‘poor glycaemic control’ (HbA1c values ≥9%).

### Convergent validity

Convergent validity was assessed using Pearson correlation between the DSMQ scores and HbA1c values. Statistical analyses of all DSMQ items were conducted by using inverted item scores, and a *p*-value of <0.05 (two-tailed test) was considered as a criterion of statistical significance for all analyses. The criteria used for interpretation of correlations were: little or no correlation = 0–0.25; fair correlation = 0.25–0.5; moderate to good correlation 0.5–0.75; and very good to excellent correlation = >0.75 [33]. The independent associations between the DSMQ subscales and HbA1c were assessed using multiple linear regression.

### Confirmatory factor analysis

Confirmatory factor analyses were performed using AMOS 21.0.0 (IBM SPSS Statistics, New York, USA) with maximum likelihood estimation. Based on the previous findings, a single-factor model of overall self-management and a four-factor model representing the four DSMQ subscales were proposed and tested. Significant correlations between the variables’ (i.e. items‘) error terms were successively modelled following relevant modification indices (threshold = 4.0). Model fit was evaluated according to the Hu & Bentler criteria [34]: Tucker Lewis Index (TLI) ≥ 0.95, Comparative Fit Index (CFI) ≥ 0.95, Root Mean Square Error of Approximation (RMSEA) ≤ 0.06 (90% CI upper bound ≤0.08). Chi^2^ statistics are additionally reported.

## Results

### Sample characteristics

The total sample consisted of 130 patients with T2DM. The demographic characteristics of the sample are displayed in the Table 1. Study participants mean age (± SD) was 51 (± 10) years, and the mean body mass index (BMI) was 30 (± 6) kg/m^2^. Female gender was slightly over represented (approx. 58%). The Majority of patients used oral hypoglycaemic agents (OHA) alone (45%) or in combination with insulin (44%), whereas 11% used insulin exclusively. The mean (± SD) duration of diabetes was 8.5 (± 7.0) years, and the mean HbA1c value was 8.6% (± 1.9). Approximately 62% of the patients had HbA1c values higher than 7.5% (59.5 mmol/mol).

**Table 1:** Characteristics of the study sample (*N* = 130)

Parameter	*n* (%) or mean ± SD
Gender	
*Male*	55 (42.4)
*Female*	75 (57.6)
Age (years)	51.3 ± 10.4
*30–45 years*	33 (25.4)
*45–60 years*	72 (55.4)
*> 60 years*	25 (19.2)
BMI (kg/m2)	29.7 ± 6.2
Education	
*No Formal Education*	50 (38.5)
*Primary level*	13 (10)
*Secondary level*	33 (25.4)
*University Level*	34 (26.2)
Diabetes duration (years)	8.5 ± 7.0
Anti-diabetic therapy	
*Exclusively insulin*	14 (10.8)
*Insulin combined with oral hypoglycemic agents*	57 (43.9)
*Oral Hypoglycemic agents only*	59 (45.3)
HbA1c value (%)	8.6 ± 1.9
DSMQ ‘Sum Scale’	4.8 ± 2.6
*Subscale ‘Glucose Management’*	5.3 ± 2.9
*Subscale ‘Dietary Control’*	4.8 ± 2.8
*Subscale ‘Physical Activity’*	4.0 ± 3.1
*Subscale ‘Health-Care Use’*	5.0 ± 2.6

Data are *n* (%) or M ± SD

*BMI* Body Mass Index, *HbA1c* glycated haemoglobin, *DSMQ* Diabetes Self-Management Questionnaire, *M* mean, *SD* standard deviation

### Psychometric properties of the Urdu version of DSMQ

#### Item characteristics and reliability

The mean (± SD) inter-item correlation was 0.76 (± 0.09). The mean item-subscale correlations were 0.79 (± 0.04) for Glucose Management, 0.76 (± 0.09) for Dietary Control, 0.77 (± 0.03) for Physical Activity, and 0.67 (± 0.19) for Health-Care Use. The overall Cronbach’s α for the Urdu version of DSMQ scale was 0.96, and it could not be increased by deleting any item. All 16 items were significantly negatively correlated with HbA1c with a mean correlation of −0.57 (± 0.06) (*p* < 0.001).

Reliability analyses revealed excellent internal consistencies of the ‘Sum Scale’ and the ‘Glucose Management’ subscale (Cronbach’s α coefficient 0.96 and 0.91). Good consistencies were observed for ‘Dietary Control’ (0.89) and ‘Physical Activity’ (0.89), whereas the ‘Health-Care Use’ subscale showed acceptable consistency (0.73).

#### Factor validity

**Fig. 2:**
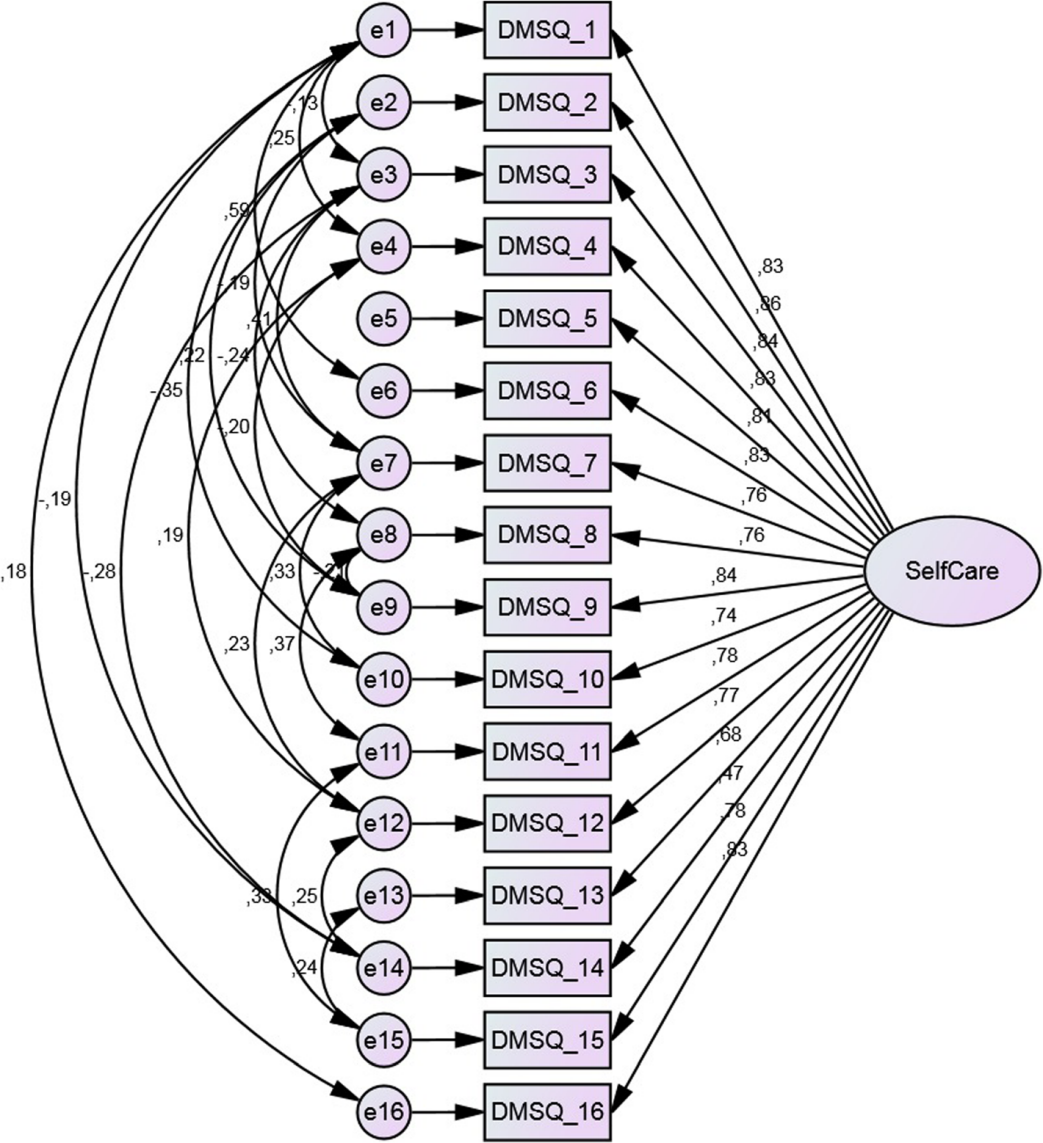
Confirmatory factor analysis a single factor model of 16 item DSMQ. Data are standardised regression coefficients for path arrows and correlation coefficients for double arrows. Boxes indicate manifest measurement variables; ovals indicate latent variables. All regression coefficients are significant with *p* < 0.001. All correlations between error terms are significant with *p* < 0.05

Factor validity was analysed using confirmatory factor analyses. First, a single factor model reflecting the sum scale was modelled. The raw model showed unsatisfactory model fit to the data: Chi^2^ = 368.8, df = 104, *p* < 0.001; TLI = 0.84; CFI = 0.86; RMSEA = 0.14 (90% CI 0.13–0.16). After successively modelling all significant correlations between the items’ error terms, adequate fit to the data was achieved, with Chi^2^ = 106.6, df = 84, *p* = 0.049; TLI = 0.98, CFI = 0.99 and RMSEA = 0.05 (90% CI 0.01–0.07). The final model is displayed in Fig. 2.

**Fig. 3:**
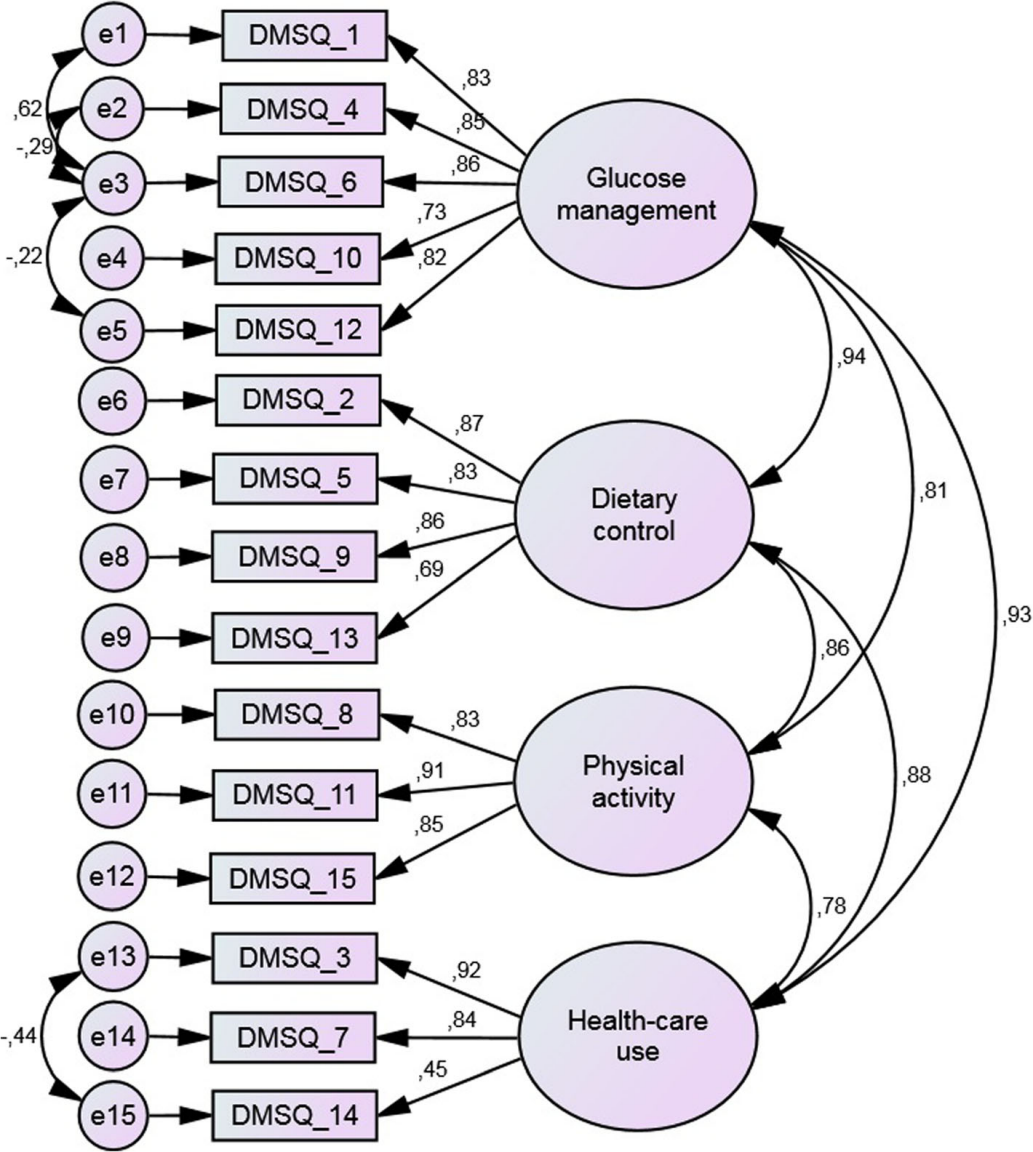
Confirmatory factor analysis a four factor model of 16 item DSMQ. Data are standardised regression coefficients for path arrows and correlation coefficients for double arrows. Boxes indicate manifest measurement variables; ovals indicate latent variables. All regression coefficients are significant with *p* < 0.001. All correlations between error terms are significant with *p* < 0.05

To test the hypothesised four-factorial structure of the DSMQ, we also modelled a model of four correlated factors representing the four scales and corresponding items. Model fit of the initial raw model was unsatisfactory (Chi^2^ = 252.6, df = 84, *p* < 0.001; TLI = 0.88; CFI = 0.90; RMSEA = 0.13 (90% CI 0.10–0.14)). After modelling significant correlations between related items’ error terms, fit was improved; however, the fit indices (Chi^2^ = 177.4, df = 80, *p* < 0.001; TLI = 0.93, CFI = 0.94, RMSEA = 0.09 (90% CI 0.08–0.11)) remained below the criteria for optimal model fit, suggesting lower fit to the data than observed for the single-factor model. Detailed results are displayed in Fig. 3.

#### Known-groups validity

Significant differences were observed between DSMQ scores of patient groups stratified according to ‘good’, ‘medium’ and ‘poor glycaemic control’. The results showed that the patients with ‘good glycaemic control’ (HbA1c values ≤7.5%) reported significantly better (*p* < 0.001) Glucose Management, Dietary Control, Physical Activity and Health-Care Use, compared to patients with ‘medium glycaemic control’ (HbA1c from 7.6% – 8.9%) and ‘poor glycaemic control’ (HbA1c values ≥9%). Correspondingly, patients with ‘good glycaemic control’ (HbA1c ≤ 7.5%) scored significantly higher on the DSMQ sum scale (7.6 ± 1.2) compared to those with ‘medium control’ (3.6 ± 1.7) and ‘poor control’ (2.8 ± 1.6).

Compared to the ‘poor glycaemic control’ group, patients with ‘medium control’ reported no significant differences in the DSMQ sum scale as well as the subscales, except for ‘Dietary Control’, where patients with ‘medium control’ scored significantly higher (*p* < 0.05) as compared to those with ‘poor control’. Complete results are shown in Table 2.

**Table 2:** Comparison of the DSMQ self-care activities in patients with HbA1c ≤ 7.5%, from 7.6 to 8.9% and ≥9.0%

DSMQ	HbA1c ≤ 7.5%	Sign.^a^	HbA1c 7.6–8.9%	Sign.^b^	HbA1c ≥ 9.0%	Sign.^c^	ANOVA
self-care activities	(*n* = 49)		(*n* = 35)		(*n* = 46)		*P*-value
Glucose Management	8.18 ± 1.46	‡	4.04 ± 1.71	ns	3.23 ± 2.22	‡	< 0.001
Dietary Control	7.72 ± 1.30	‡	3.62 ± 1.88	*	2.74 ± 1.55	‡	< 0.001
Physical Activity	6.96 ± 1.97	‡	2.79 ± 2.35	ns	1.81 ± 1.86	‡	< 0.001
Health-Care Use	7.14 ± 1.62	‡	4.03 ± 2.22	ns	3.45 ± 1.99	‡	< 0.001
Sum Scale	7.64 ± 1.16	‡	3.63 ± 1.74	ns	2.81 ± 1.58	‡	< 0.001

Data are M ± SD. Tests were One-way ANOVA and Scheffé Test for post-hoc group comparisons. Scheffé Test significance is expressed: *p < 0.05; ‡*p* < 0.001; ns, not significant

DSMQ, Diabetes Self-Management Questionnaire; HbA1c, glycated haemoglobin; ANOVA, Analysis of Variance

^a^regards comparison between the first and second group

^b^regards comparison between the second and third group

^c^regards comparison between the third and first group

The Spearman correlation between HbA1c and DSMQ sum scale was −0.78 (*p* < 0.001), indicating a high inverse association between the two. Likewise, the four DSMQ subscales also showed significant inverse correlations with HbA1c, as shown in Table 3.

**Table 3:** Correlations of the DSMQ scales with patient characteristics and HbA1c

	DSMQ scales				
	*GM*	*DC*	*PA*	*HU*	*SS*
Gender	−0.11	−0.04	−0.02	−0.10	−0.08
BMI	−0.05	−0.09	−0.09	−0.06	−0.07
Age	0.00	0.10	0.01	−0.07	0.02
Diabetes Duration	0.13	0.09	0.10	0.05	0.11
HbA1c	−0.75‡	−0.76‡	−0.71‡	−0.64‡	−0.78‡

Coefficients are Spearman’s ρ; ‡ *P* < 0.001 (two-tailed test)

Coefficients which represent type 2 diabetes patients (*n* = 130) are DSMQ, Diabetes Self-Management Questionnaire; BMI, Body Mass Index; HbA1c, glycated haemoglobin; GM, Glucose Management; DC, Dietary Control; PA, Physical Activity; HU, Health-Care Use; SS, Sum Scale

The associations between the DSMQ scales and HbA1c were also assessed in a single multiple regression analysis which revealed that when assessed together only the Dietary Control and Glucose Management subscales were significantly associated with better glycaemic control (Beta = −0.29, *p* = 0.028 and Beta = −0.42, *p* = 0.004, respectively), while the Physical Activity and Health-Care Use subscales were not (Beta = −0.18, *p* = 0.09 and Beta = 0.13, *p* = 0.28, respectively), as shown in Table 4.

**Table 4:** Linear associations of the DSMQ sub-scales with HbA1c in type 2 diabetes patients (*N* = 130)

Predictors	Beta [95% CI]	*p*-value
Dietary control	−0.30 [−0.38 – −0.02]	0.028
Glucose management	−0.42 [−0.46 – −0.09	0.004
Physical activity	−0.18 [−0.24–0.02]	0.088
Health-care use	0.13 [−0.08–0.27]	0.282

## Discussion

The main objective of this study was to evaluate the psychometric properties of the newly developed Urdu version of the DSMQ using a convenience sample of patients with type 2 diabetes in Pakistan. This study was the first to systematically translate and validate the sixteen-item DSMQ in Urdu. Conceptually comparable to the original DSMQ, which was tested [23] on German patients with type 1 and type 2 diabetes, we found to have very good psychometric properties with high reliability and very good convergent properties as well as factorial validity.

Overall internal consistency (Cronbach’s α) for the Urdu version of DSMQ in our study among Pakistani type 2 diabetes was excellent, in fact even better than that was found in the study by Schmitt et al. [23]. Likewise, consistencies of the subscales were also very good, and comparatively better than those observed by Schmitt et al. (GM: 0.77; DC: 0.77; PA: 0.76; HU: 0.60) [23]. A comparatively high mean item-total correlation was observed in our study. Unlike Germany, where DSMQ was originally developed and validated, in current study, which was conducted in Pakistan, the health care infrastructure and facilities are comparatively less in comparison to the developed countries, which could be one of the major factor for lower mean items scores for Health-Care Use sub-scle.

Glycemic control as assessed by HbA1c, was found significantly correlated to all DSMQ scores, at which good glycemic control (HbA1c ≤ 7.5%) was significantly associated with higher scores on the DSMQ sum scale as well its subscales. This finding supports the fact that those patients who practice good self-care management and are more concerned about their disease have good glycaemic control and experience lesser chances of diabetes associated late complications [7–9]. A low prevalence of diabetes associated neuropathy and nephropathy has been reported in T2DM patients in Iran, who scored higher on Glucose Management scale of DSMQ [35].

All DSMQ subscales showed high correlations with HbA1c. However, the linear regression showed that only the Glucose Management and Dietary Control subscales were independently associated with HbA1c, whereas Physical Activity and Health-Care Use were not. In the original DSMQ study [23], patients with good glycaemic control (HbA1c ≤ 7.5%), reported higher score for Physical Activity and Glucose Management, which could be due to cultural and health care facilities difference among the countries. More or less a similar finding was observed in a recent study conducted in Iran [35], where higher scores on Glucose Management and Dietary Control subscales were reported as compared to rest of the sub-scales of the DSMQ.

The known-groups comparison analysis support the Urdu version of the DSMQ as a valid instrument for measuring self-care behaviours related to glycaemic control, as the instrument was able to differentiate between patients with different levels of glycemic control. This finding is in line with our hypotheses as patients with higher DSMQ scores were expected to perform better self-care behaviour and thus have better metabolic control. The tool will be helpful in identifying the problems regarding self-care activities. The strong associations between self-care behaviours as assessed with the Urdu DSMQ suggest that educating and helping patients improve their diabetes self-care behaviours, especially related to Glucose Management and Dietary Control might lead to improved glycaemic control and reduced risks of complications.

## Conclusion

The Urdu version of the DSMQ was found to be a reliable and valid instrument for measuring self-care behaviours in type 2 diabetes patients in Pakistan. Results for the Urdu version are comparable to those of the original study. The Urdu version of DSMQ can be of significant value for clinical studies examining glycaemic control in relation to patients’ diabetes self-care behaviours as well as for clinicians seeking to assess their patients’ self-care in order to identify behaviours in need of improvement.

### Strength and limitations

This study is the first to translate and validate the DSMQ in Urdu language. A possible limitation of this study is that it was used to assess self-care behaviours in type 2 diabetes patients; diabetes type 1 patients should also be recruited to establish validity and reliability of this tool across both major diabetes types. Low literacy rate of the respondents is another limitation of this study, as it might limit generalisability to other patient groups. However, the results of this study could be generalised to the community as the data were collected from the institutional diabetes care clinics, which cater the needs of majority of diabetes patients. The strength of this study lie in the standardised data assessment using structured interviews and HbA1c analysis in one laboratory.

## Additional files

Additional file 1: English version of Diabetes Self-Management Questionnaire (DSMQ). (PDF 11 kb)

Additional file 2: Urdu version of Diabetes Self-Management Questionnaire. (PDF 158 kb)

## Abbreviations

DC: Dietary Control
DM: Diabetes Mellitus
DSMQ: Diabetes Self-Management Questionnaire
GM: Glucose Management
HbA1c: Glycosylated Haemoglobin
HU: Health-Care Use
PA: Physical Activity
SDSCA: Summary of Diabetes Self-Care Activities
