# Supplementary material for: Psychometric properties of the Diabetes Self-Management Questionnaire (DSMQ) in Urdu
Source: Health Qual Life Outcomes. 2017 Oct 12;15:200. doi: 10.1186/s12955-017-0776-8 (PMC5639758; doi:10.1186/s12955-017-0776-8)
Supplement: Supplementary file 1 — English version of Diabetes Self-Management Questionnaire (DSMQ). (PDF 11 kb) [file 12955_2017_776_MOESM1_ESM.pdf]

Diabetes Self-Management Questionnaire (DSMQ)

The following statements describe self-care activities related to your diabetes. Thinking about your self-care over the **last 8 weeks**, please specify the extent to which each statement applies to you.	applies to me very much	applies to me to a consider-able degree	applies to me to some degree	does not apply to me
1. I check my blood sugar levels with care and attention.<br>☐ *Blood sugar measurement is not required as a part of my treatment.*	☐3	☐2	☐1	☐0
2. The food I choose to eat makes it easy to achieve optimal blood sugar levels.	☐3	☐2	☐1	☐0
3. I keep all doctors' appointments recommended for my diabetes treatment.	☐3	☐2	☐1	☐0
4. I take my diabetes medication (e. g. insulin, tablets) as prescribed.<br>☐ *Diabetes medication/insulin is not required as a part of my treatment.*	☐3	☐2	☐1	☐0
5. Occasionally I eat lots of sweets or other foods rich in carbohydrates.	☐3	☐2	☐1	☐0
6. I record my blood sugar levels regularly (or analyse the value chart with my blood glucose meter).<br>☐ *Blood sugar measurement is not required as a part of my treatment.*	☐3	☐2	☐1	☐0
7. I tend to avoid diabetes-related doctors' appointments.	☐3	☐2	☐1	☐0
8. I do regular physical activity to achieve optimal blood sugar levels.	☐3	☐2	☐1	☐0
9. I strictly follow the dietary recommendations given by my doctor or diabetes specialist.	☐3	☐2	☐1	☐0
10. I do not check my blood sugar levels frequently enough as would be required for achieving good blood glucose control.<br>☐ *Blood sugar measurement is not required as a part of my treatment.*	☐3	☐2	☐1	☐0
11. I avoid physical activity, although it would improve my diabetes.	☐3	☐2	☐1	☐0
12. I tend to forget to take or skip my diabetes medication (e. g. insulin, tablets).<br>☐ *Diabetes medication/insulin is not required as a part of my treatment.*	☐3	☐2	☐1	☐0
13. Sometimes I have real 'food binges' (not triggered by hypoglycaemia).	☐3	☐2	☐1	☐0
14. Regarding my diabetes care, I should see my medical practitioner(s) more often.	☐3	☐2	☐1	☐0
15. I tend to skip planned physical activity.	☐3	☐2	☐1	☐0
16. My diabetes self-care is poor.	☐3	☐2	☐1	☐0

© Dr Andreas Schmitt, Research Institute of the Diabetes Academy Mergentheim, Germany, 2012
