# Supplementary material for: Psychometric properties of the Diabetes Self-Management Questionnaire (DSMQ) in Urdu
Source: Health Qual Life Outcomes. 2017 Oct 12;15:200. doi: 10.1186/s12955-017-0776-8 (PMC5639758; doi:10.1186/s12955-017-0776-8)
Supplement: Supplementary file 2 — Urdu version of Diabetes Self-Management Questionnaire. (PDF 158 kb) [file 12955_2017_776_MOESM2_ESM.pdf]

ذیابطیس کی ذاتی دیکھ بھال کا سوالنامہ (DSMQ)

	مندرجہ ذیل بیانات آپ کی ذیابطیس کی ذاتی دیکھ بھال کی سر گرمیوں کے متعلق ہیں۔ **<u>گذشتہ ا ٓٹھ (8) ہفتوں تک</u>** کی اپنی تمام ذاتی دیکھ بھال کے بارے میں سوچتے ہوئے،براہ مہربانی جو بیان آپ پر جس حد تک لاگو ہوتا ہو،اس کی نشاندہی کریں۔	مجھ پر لاگو ہوتا / ہوتی ہے			
		**بہت زیادہ**	**کافی حد تک**	**کچھ حد تک**	**بالکل نہیں**
1	میں اپنے خون میں شوگر کی سطح کو توجہ اور احتیاط سے جانچتا / جانچتی ہوں ۔<br>□ خون میں شوگر کی سطح کو جانچنا میرے علاج کے کے لیے ضروری **<u>نہیں</u>** ہے۔	□3	□2	□1	□0
2	خوراک کا انتخاب میرے خون میں شوگر کی سطح کا بہتر معیار حاصل کرنے میں آسانی پیدا کرتا / کرتی ہے۔	□3	□2	□1	□0
3	میں ڈاکٹروں سے اپنے ذیابطیس کے علاج کے لیے تجویز کردہ ملاقاتوں (دن اور وقت) کے مطابق ملتا ہوں ۔	□3	□2	□1	□0
4	میں اپنی ذیابطیس کے لیے ہدایت کردہ ادویات (جیسے انسولین اور گولیاں وغیرہ) استعمال کرتا / کرتی ہوں۔<br>□ ذیابطیس کی ادویات / انسولین میرے علاج کے لیے ضروری **<u>نہیں</u>** ہیں۔	□3	□2	□1	□0
5	کبھی کبھار میں بہت سی مٹھائیاں اور کثیر مقدار میں دوسری نشاستہ دار (کاربوہائیڈریٹ والی) خوراک کھاتا / کھاتی ہوں۔	□3	□2	□1	□0
6	میں اپنے خون میں شوگر کی سطح کو باقاعدگی سےنوٹ کرتا / کرتی ہوں۔<br>□ خون میں شوگر کی سطح کو جانچنا میرے علاج کے لیے ضروری **<u>نہیں</u>** ہے۔	□3	□2	□1	□0
7	میں ذیابطیس کے متعلق ڈاکٹروں کی تجویز کردہ ملاقاتوں (دن اور وقت) کو نظر انداز کرتا / کرتی ہوں۔	□3	□2	□1	□0
8	میں خون میں شوگر کی بہترسطح کو حاصل کرنے کے لیے با قاعدگی سے جسمانی سرگرمی کرتا / کرتی ہوں۔	□3	□2	□1	□0
9	میں ڈاکٹروں یا ماہرذیابطیس کی دی گئی غذائی تجاویز پر سختی سے عمل کرتا / کرتی ہوں۔	□3	□2	□1	□0
10	میں اپنے خون میں شوگر کی سطح کو اتنا متواتر چیک **<u>نہیں</u>** کرتا / کرتی ,جتنا کہ خون میں گلوکوز کی مناسب سطح کو حاصل کرنے کے لیے ضروری ہوتا ہے ۔<br>□ خون میں شوگر کی سطح کو جانچنا میرے علاج کے کے لیے ضروری **<u>نہیں</u>** ہے۔	□3	□2	□1	□0
11	میں جسمانی سرگرمی کو نظر انداز کرتا / کرتی ہوں، حالانکہ یہ میری ذیابطیس کے کنٹرول کو بہتر بنا تی ہے۔	□3	□2	□1	□0
12	میں ذیابطیس کی ادویات چھوڑدیتا / دیتی یا لینا بھول جاتا / جاتی ہوں۔<br>□ ذیابطیس کی ادویات /انسولین میرے علاج کے لیے ضروری نہیں ہیں۔	□3	□2	□1	□0
13	بعض اوقات مجھے بہت زیادہ بھوک محسوس ہوتی ہے۔ (جو کہ خون میں شوگر کی سطح کی کمی کی وجہ سے **<u>نہیں</u>** ہے)	□3	□2	□1	□0
14	مجھے اپنی ذیابطیس کی دیکھ بھال کے متعلق اپنے ڈاکٹروں کے پاس کثرت سے جانا چاہئے۔	□3	□2	□1	□0
15	میں اپنی طے شدہ جسمانی سرگرمیاں چھوڑ دیا کرتا / کرتی ہوں۔	□3	□2	□1	□0
16	میری ذیابطیس کی ذاتی دیکھ بھال بہت بری ہے۔	□3	□2	□1	□0

Translated and Adapted by Allah Bukhsh & Tahir Mehmood Khan (allah.bukhsh@monash.edu, tahir.mehmood@monash.edu)
School of Pharmacy, MONASH University, Malaysia, 2016
© Dr Andreas Schmitt, Research Institute of the Diabetes Academy Mergentheim, Germany, 2012
